# Supplementary material for: Establishing the Reliability of the GaitON® Motion Analysis System: A Foundational Study for Gait and Posture Analysis in a Healthy Population
Source: Sensors (Basel). 2024 Oct 26;24(21):6884. doi: 10.3390/s24216884 (PMC11870036; doi:10.3390/s24216884)
Supplement: Supplementary file 1 [file sensors-24-06884-s001.zip › sensors-3223949-supplementary.pdf]

GaitON® | by auptimo

MARKERS FOR WALKING GAIT ANALYSIS

LATERAL VIEW

S No.	Marker Name	Type of Marker
1	Greater trochanter of the femur	Flat
2	Lateral epicondyle of the femur	Flat
3	Lateral malleolus	Flat
4	Line parallel to the sole of the foot<br>Barefeet : Marker 1 and 2 in Figure 2<br>Shod : Marker 1 and 2 in Figure 3	Flat

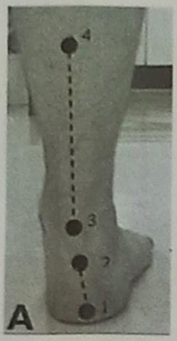

Figure 1

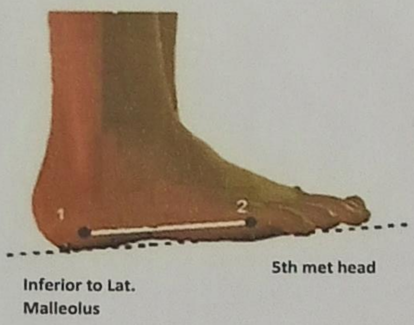

Figure 2

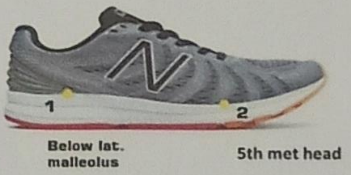

Figure 3

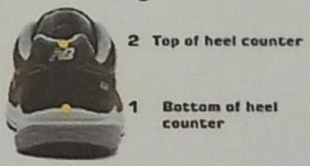

Figure 4

POSTERIOR VIEW

S No.	Marker Name	Type of Marker
1	Posterior Superior Iliac Spine(PSIS's)	Flat
2	Base of the calcaneus<br>Barefeet : (Marker 1 in Figure 1)<br>Shod: (Marker 1 in Figure 4)	Flat/ Black Dot
3	Achilles tendon attachment (Figure 1)<br>Barefeet : (Marker 2 in Figure 1)<br>Shod: (Marker 2 in Figure 4)	Flat/ Black Dot
4	Centre of Achilles tendon at the height of the medial malleolus (Marker 3 in Figure 1)	Flat/ Black Dot
5	Centre of the posterior aspect of the shank approx. 15 cm above marker 3 (Marker 4 in Figure 1)	Flat/Black Dot

ANTERIOR VIEW

S No.	Marker Name	Type of Marker
1	Centre point of the patella	Flat

Type of marker

Ball: Hemispherical foam based markers

Black Dot: Temporary black pen

Flat: Flat and circular sticker

08/09/2024 18:55

GaitON® | by auptimo

MARKERS FOR POSTURE ANALYSIS

LATERAL VIEW

S No.	Marker Name	Type of marker
1	C7 spinous process (Figure 1)	Ball
2	Mid-Point of the humeral head (Figure 1)	Ball
3	Greater trochanter of the femur	Flat
4	Lateral epicondyle of the femur	Black Dot / Flat
5	Lateral malleolus	Black Dot / Flat

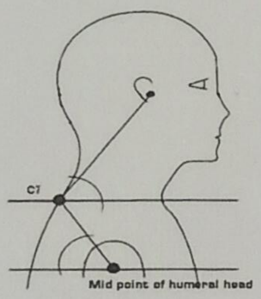

Figure 1

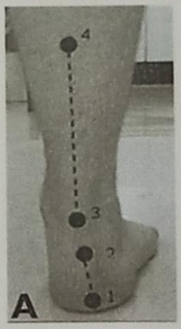

Figure 2

POSTERIOR VIEW

S No.	Marker Name	Type of marker
1	Base of the calcaneus (Marker 1 in Figure 2)	Flat/ Black Dot
2	Achilles tendon attachment (Marker 2 in Figure 2)	Flat/ Black Dot
3	Centre of Achilles tendon at the height of the medial malleolus (Marker 3 in Figure 2)	Flat/ Black Dot
4	Centre of the posterior aspect of the shank approx. 15 cm above marker 3 (Marker 4 in Figure 2)	Flat/Black Dot

ANTERIOR VIEW

S No	Marker Name	Type of marker
1	Anterior Superior Iliac Spine(ASIS's)	Flat
2	Centre point of the patella	Flat /Black Dot
3	Tibial Tuberosity (For measuring Q angle)	Flat /Black Dot

Type of marker

Ball: Hemispherical foam based markers

Black Dot: Temporary black pen

Circle: Flat and circular sticker

08/09/2024 18:55
